# Selection of bone graft type for the surgical treatment of thoracolumbar spinal tuberculosis based on the spinal instability neoplastic score: a retrospective single-center cohort study

**DOI:** 10.1186/s12891-023-06620-6

**Published:** 2023-06-24

**Authors:** Tianji Huang, Zhenghan Han, Wei Luo, Bin He, Yong Zhu, Zenghui Zhao

**Affiliations:** 1 Department of Orthopedic Surgery, The First Affiliated Hospital of Chongqing Medical University, Chongqing, 400016 People’s Republic of China; 2 Orthopedic Laboratory of Chongqing Medical University, Chongqing, 400016 People’s Republic of China

**Keywords:** Thoracic vertebrae, Lumbar vertebrae, Tuberculosis, Spinal, Bone transplantation, Surgical procedures, Operative

## Abstract

**Objectives:**

This study aimed to establish a standard for selecting bone graft type for thoracolumbar spinal tuberculosis surgery based on the spinal instability neoplastic score (SINS).

**Methods:**

Patients with thoracolumbar tuberculosis who underwent one-stage debridement posteriorly and instrumentation were divided into a structural bone graft group (SBG) (51 cases) and a non-structural bone graft group (NSBG) (54 cases) according to their SINS. SBG was performed when the SINS was ≥ 13 and NSBG was performed when it was 7 ≤ SINS ≤ 12. Baseline data, clinical outcomes, and imaging outcomes were collected and statistically analyzed between the two groups.

**Results:**

Significant improvements in clinical and imaging outcomes were achieved in both groups. Compared to the SBG group, the operation time of the NSBG group was shorter, the intraoperative blood loss of the NSBG group was less, the bone fusion time of the NSBG group was faster.

**Conclusion:**

Non-structural and structural bone grafting can achieve comparable therapeutic effects in patients with spinal tuberculosis, and a suitable selection of bone grafts based on quantitative SINS will make full use of the advantages of different bone grafts.

## Introduction

Spinal tuberculosis is the most common form of extrapulmonary tuberculosis, accounting for approximately half of all cases of skeletal tuberculosis [1]. Spinal tuberculosis typically destroys the intervertebral discs and adjacent vertebrae, leading to spinal instability and kyphosis [2–4]. When surgery is indicated, the affected vertebral bone and disc should be removed, which always requires bone grafting. Many types of bone grafts have been successfully reported, and are mainly divided into non-structural and structural types, including the ilium, titanium mesh, and rib bone [5–7]. However, precise criteria for selecting the appropriate bone graft type are lacking [8]. Spinal instability is a surgical indication for many spinal disorders, such as spinal tuberculosis, and many criteria have been proposed and reported. However, there is no consensus regarding the criteria for evaluating the stability of tuberculosis-infected spinal columns. The spinal instability neoplastic score (SINS) has been widely used to evaluate the stability of the tumor-involved spine by assessing six factors: location, mechanical pain, bone lesion quality, spinal alignment, vertebral body collapse, and posterolateral involvement of spinal elements [9]. Inspired by the SINS, we aimed to use it to evaluate the spinal stability of tuberculosis-infected spines.

The objective of the present retrospective cohort study was to assess the results of patients with thoracolumbar tuberculosis who underwent reconstruction using non-structural or structural bone grafts after one-stage posterior debridement and internal fixation determined by SINS, to establish a rule for selecting bone grafts for spinal reconstruction and fusion of thoracolumbar tuberculosis.

## Materials and methods

The Ethics Committee of The First Affiliated Hospital of Chongqing Medical University approved this study (2019 − 123), and informed consent was obtained from all patients in this research. All methods were carried out in accordance with relevant guidelines and regulations.

### Selection of patients

Between 2016 and 2020, 105 patients who were diagnosed with thoracolumbar tuberculosis and underwent posterior surgery were retrospectively reviewed. The selection criteria were as follows: (1) pathologically confirmed thoracolumbar spinal tuberculosis; (2) single-segment tuberculosis; (3) surgical treatment with one-stage posterior debridement, bone graft fusion, and instrumentation; (4) a structural bone graft (SBG) (titanium mesh bone graft) or non-structural bone graft (NSBG) (autologous granular bone graft) was used in the procedure; (5) the follow-up time was no less than two years; and (6) all required data had been collected completely. The exclusion criteria were as follows: (1)spinal tuberculosis involving the cervical spine; (2) patients who have previously undergone spinal procedures; and (3) patients with primary or metastatic tumors and active pulmonary tuberculosis.

### Preoperative management

Four anti-tuberculosis drugs (isoniazid 0.3 g qd; rifampicin 0.45 g qd; pyrazinamide 1.5 g qd; and ethambutol 0.75 g qd) were used for 2–4 weeks before the operation. Toxic manifestations of tuberculosis were alleviated, and the patients’ comorbidities were controlled preoperatively.

### Operative procedure

All patients received general anesthesia. A longitudinal midline incision was made in the prone position to expose the posterior elements of the spine. One or two normal vertebral bodies above and below the lesion segments were instrumented using suitable posterior pedicle screws. Temporary stability was maintained using a rod that was fixed on the side of the debridement. Unilateral laminectomy or pedicle resection was performed to remove the necrotic tissue, bone fragments, and necrotic discs.

**Fig. 1:**
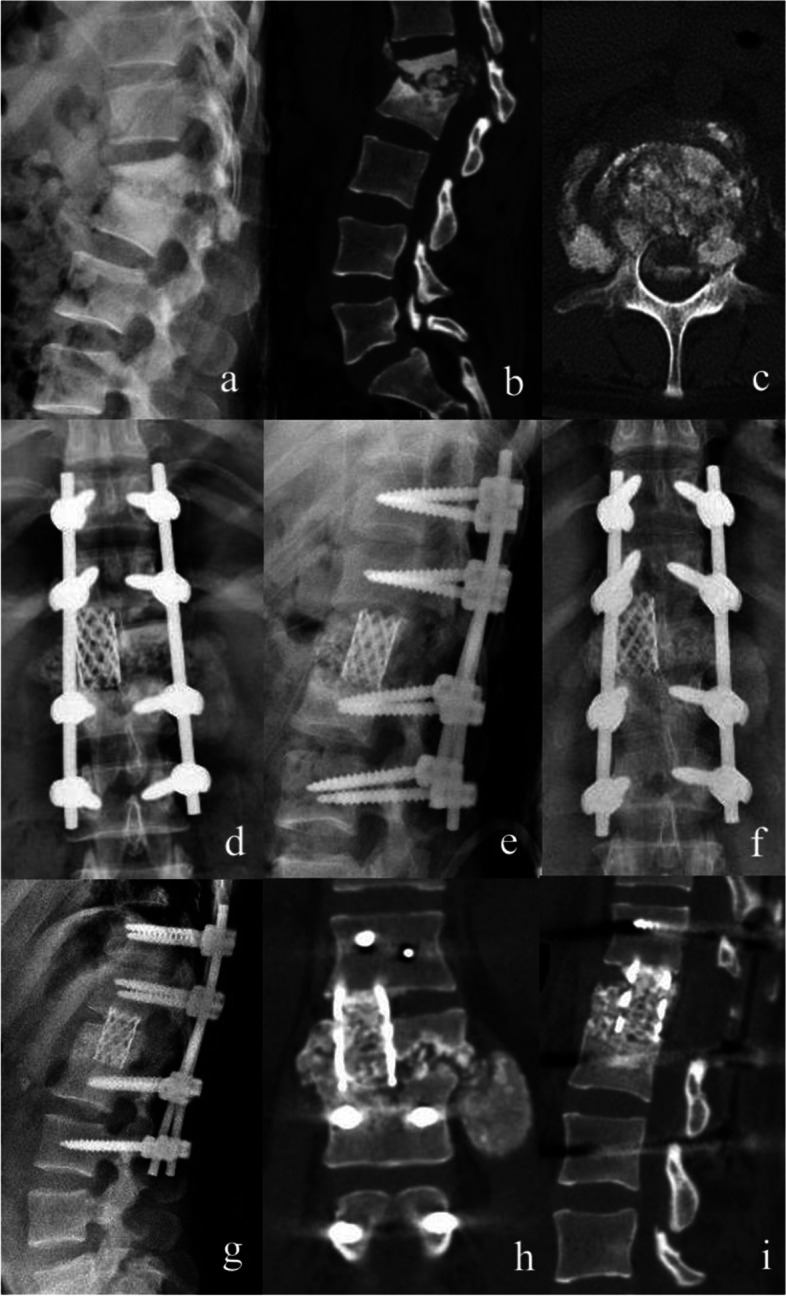
A 31 years old female diagnosed as L1-2 tuberculosis and treated with structural bone graft. **a**-**c** Preoperative X-ray and CT scan. **d**-**e** Postoperative X-ray. **f**-**i **X-ray and CT scan at last follow-up

**Fig. 2:**
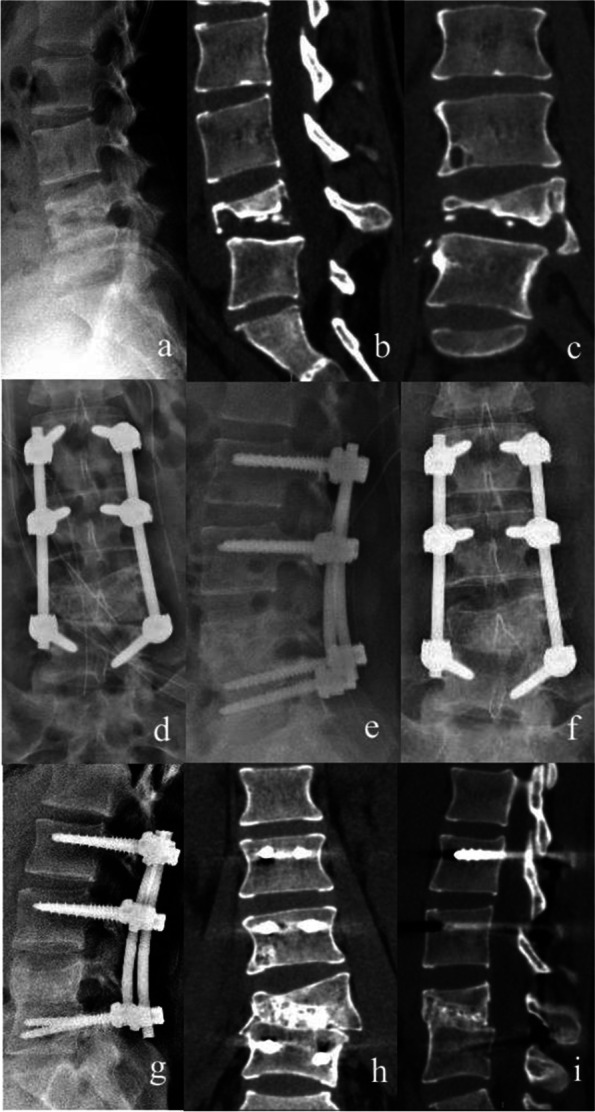
A 22 years old female diagnosed as L4-5 tuberculosis and treated with non-structural bone graft. **a**-**c** Preoperative X-ray and CT scan. **d**-**e** Postoperative X-ray. **f**-**i** X-ray and CT scan at last follow-up

SBG was performed when SINS was ≥ 13 and NSBG was performed when 7 ≤ SINS ≤ 12. In the SBG group (Fig. 1), a sizable titanium mesh filled with crushed granular bone particles and 1.0 g of streptomycin was implanted into the vertebra. In the NSBG group (Fig. 2), the vertebral body was implanted with granular bones from the spinous process and vertebral plate that was harvested intraoperatively. The posterior part of the vertebral body was covered by a gelatin sponge containing isoniazid when granular bones entered the spinal canal. After washing with enough normal saline, isoniazid 0.3 g and streptomycin powder 1.0 g were placed in the surgical site for local treatment. Finally, the rods were locked and the surgical incision was sutured.

### Postoperative management

Antibiotics were administered within 3 days to prevent infection. The drainage tube removal criterion was a drainage volume of less than 40 mL/day. The patients were treated with the same anti-tuberculosis drugs for 18–24 months. Braces were used for 3–6 months after surgery. A high-protein nutritional diet was provided throughout the treatment period. Routine blood examinations, erythrocyte sedimentation rate (ESR), C-reactive protein (CRP), liver function, kidney function, X-ray, computed tomography, and magnetic resonance imaging, when necessary, were performed during follow-up at 1, 3, 6, and 12 months postoperatively.

### Follow-up index

The operation time, bleeding volume, and length of hospital stay were recorded. Visual analog scale (VAS), Oswestry disability index (ODI), ESR, CRP, and American Spinal Injury Association (ASIA) grade of neurological function were assessed before surgery and at the last follow-up. The criterion of Bridwell et al. was used to evaluate the bone graft fusion time according to the results of computed tomography scans during each follow-up [10]. The four grades were described by Bridwell et al. [10]. In the present study, bone graft fusion was defined as grade I or II. The correction of the Cobb angle was defined as the difference between post- and pre-operative Cobb angles. Loss of Cobb angle was defined as the difference between the last follow-up Cobb angle and the postoperative Cobb angle.

### Statistical analysis

SPSS 21.0 was used for analyzing data. The Kolmogorov–Smirnov test was used to assess if the values were normally distributed. An independent sample t-test was used to compare normally distributed continuous variables (presented as the mean ± standard deviation) between the two groups. The Mann-Whitney U test was used to compare non-normally distributed continuous variables (presented as median and range) between the two groups. The chi-squared test was used to compare categorical variables between the two groups. A matched t-test was used for intragroup comparisons of normally distributed continuous variables. The significance level was set as 0.05.

## Results

### Patients’ baseline information

During the surgical procedure, 51 and 54 patients received structural and non-structural bone grafts, respectively. The patients were followed for 24–66 months. No significant differences were found in sex, age, body mass index, paravertebral abscess percentage, or follow-up time between the SBG and NSBG groups (Table 1).

**Table 1:** Comparison of baseline data between the two groups

	Structural bone graft (*N* = 51)	Non-structural bone graft (*N* = 54)	*P* Value
Gender (male / female)^a^	24/27	31/23	0.289
Age (year)^b^	34(22–76)	35(21–75)	0.783
BMI (kg/m^2^) ^c^	21.1 ± 2.3	21.5 ± 2.5	0.353
Paravertebral abscess (yes/no) ^a^	35/16	35/19	0.679
Follow up time (month) ^b^	31 (24–66)	32 (24–63)	0.835

^a^ The categorical variables were compared by Chi-square test

^b^ The non-normally distributed continuous variables were presented as median (range) and compared by Mann-Whitney U test

^c^ The normally distributed continuous variables were presented as mean ± standard deviation and compared by independent sample t-test

### Clinical outcomes

The operation time in the SBG group was longer than that in the NSBG group (*P* = 0.000). Intraoperative blood loss in the SBG group was higher than that in the NSBG group (*P* = 0.000). The length of hospital stay did not differ significantly between the two groups. No significant differences were found in preoperative VAS and last follow-up VAS scores between the SBG and NSBG groups. The preoperative ODI of the SBG group was significantly higher than that of the NSBG group (*P* = 0.041), while no difference in the final follow-up ODI was found between the groups. Compared with the SBG group, the preoperative ESR of the NSBG group was significantly lower (*P* = 0.005), while no difference was found in the last follow-up ESR between the groups. No significant differences were found in preoperative CRP and last follow-up CRP levels between the SBG and NSBG groups (Table 2). Compared to the corresponding preoperative parameters, the VAS, ODI, ESR, and CRP were all remarkably improved in the last follow-up of each group according to results of matched t-tests (*P* = 0.000).

**Table 2:** Comparison of clinical outcomes between the two groups

	Structural bone graft (*N* = 51)	Non-structural bone graft (*N* = 54)	*P* Value
Operation time (min) ^a^	209.7 ± 31.9	175.8 ± 33.8	0.000
Intraoperative blood loss (ml) ^a^	552.9 ± 198.3	340.7 ± 122.1	0.000
Hospital stay (d) ^a^	15.0 ± 2.8	15.7 ± 2.9	0.213
Preoperative VAS ^a^	6.0 ± 1.6	5.8 ± 1.7	0.421
Last follow up VAS ^b^	1 (0–5)	1 (0–4)	0.568
Preoperative ODI ^a^	45.1 ± 14.9	38.8 ± 16.1	0.041
Last follow up ODI ^a^	16.0 ± 5.8	15.0 ± 5.8	0.381
Preoperative ESR (mm/h) ^a^	48.9 ± 14.2	41.4 ± 12.7	0.005
Last follow up ESR (mm/h) ^a^	16.4 ± 5.2	14.8 ± 5.4	0.123
Preoperative CRP (mg/L) ^a^	26.1 ± 12.6	23.9 ± 10.8	0.347
Last follow up CRP (mg/L) ^a^	6.5 ± 3.1	7.5 ± 4.3	0.163

^a^ The normally distributed continuous variables were presented as mean ± standard deviation and compared by independent sample t-test

^b^ The non-normally distributed continuous variables were presented as median (range) and compared by Mann-Whitney U test

### Imaging outcomes

No implant failure was observed at the last follow-up. Compared to the SBG group, the preoperative Cobb angle, postoperative Cobb angle, last follow-up Cobb angle, and correction of the Cobb angle were all lower in the NSBG group (*P* = 0.000, 0.000, 0.002, and 0.019, respectively). The reason may be that the SBG group patients’ kyphosis is generally more serious than that in USBG group patients. There was no significant difference between the loss of Cobb angles in both groups. The bone graft fusion time in the NSBG group was significantly shorter than that in the SBG group (*P* = 0.007) (Table 3).

**Table 3:** Comparison of imaging outcomes between the two groups

	Structural bone graft (*N* = 51)	Non-structural bone graft (*N* = 54)	*P* Value
Preoperative Cobb angle (°) ^a^	21.5 ± 6.3	14.8 ± 10.5	0.000
Postoperative Cobb angle (°) ^a^	9.2 ± 5.6	5.0 ± 6.0	0.000
Last follow-up Cobb angle (°) ^a^	11.6 ± 5.8	7.7 ± 7.1	0.002
Correction of Cobb angle (°) ^a^	12.3 ± 4.4	9.8 ± 6.3	0.019
Loss of Cobb angle (°) ^a^	2.3 ± 1.5	2.7 ± 2.4	0.419
Bone graft fusion time (month) ^b^	7 (4–18)	6 (4–12)	0.007

^a^ The normally distributed continuous variables were presented as mean ± standard deviation and compared by independent sample t-test

^b^ The non-normally distributed continuous variables were presented as median (range) and compared by Mann-Whitney U test

### Neurological function and complications

At the last follow-up in the SBG group, the ASIA scale of two patients improved from C to D, that of two patients improved from C to E, and that of nine patients improved from D to E. At the last follow-up in the NSBG group, the ASIA scale of two patients improved from C to D, that of one patient improved from C to E, and that of 14 patients improved from D to E.

In the SBG group, 13 patients (25.5%) had complications: one patient had leakage of cerebrospinal fluid, two had kidney function lesions, three had liver function lesions, one had a urinary tract infection, two had pulmonary infections, two had deep venous thrombosis, and two had sinus formation. In the NSBG group, 15 patients (27.8%) had complications, including two cases of liver function lesions, two of kidney function lesions, three of deep venous thrombosis, two of sinus formation, three of urinary tract infections, and three of pulmonary infections. According to the chi-square test, the number of postoperative complications was comparable between the SBG and NSBG groups (P = 0.791). After conservative treatment, all complications resolved without serious consequences.

## Discussion

Typically, spinal tuberculosis always destroys the intervertebral disc and deconstructs the spinal bone, leading to spinal instability. Surgical management of spinal tuberculosis includes complete debridement, decompression of neurological deficits, correction of deformities, bone grafting, and stable internal fixation to achieve solid fusion [11]. Several types of bone grafts, which are mainly categorized as non-structural or structural grafts, have been successfully used for intervertebral reconstruction and fusion, including the iliac bone, titanium mesh, and rib bone [5–7]. However, the type of bone graft that should be properly selected during surgery remains controversial.

Many studies have reported the merits of using a titanium mesh as a dependable reconstruction method for satisfactory sagittal profile maintenance and bone fusion rate, with fewer problems in implants [12]. Titanium mesh eliminates the need of harvesting iliac bone as the adequate resource of local bone either from lamina or spinal process, decreasing donor site complications. However, it is technically demanding to insert a titanium mesh into the intervertebral space, and in this process, the operation time and the incidence of nerve injury will increase [13]. Autogenous bone harvested from the iliac crest is the gold standard for bone defect repair owing to its good biocompatibility, bone conductibility, and osteogenesis [14]. Several studies [15–18] have reported that NSBGs could reduce surgical difficulty, surgical injury, and procedure time, and the outcomes of NSBGs are similar to those of titanium mesh with autogenous bone in reconstruction of the spine. In addition, NSBGs with granular bone can be placed into the intervertebral space more easily than titanium mesh cage, and is associated with a lower incidence of nerve injury. A meta-analysis by He et al. also supported this view and declared that titanium meshes did not show an advantage, as reported by previous studies, when considering imaging outcomes, effectiveness, or surgical complications [19].

The Denis three-column theory and loading-sharing principles may be violated when using the NSBG method [20, 21]. However, the use of NSBG in spinal tuberculosis has been proven to be successful in a few studies [16–18]. Our previous research also revealed that NSBG had satisfactory clinical and imaging outcomes when used to treat tuberculosis involving a single-segment of the thoracic spine compared to the outcomes of cases treated with the SBG method [15]. Owing to the rigidity of pedicle screw placement, the use of NSBG in our study did not result in instrumentation failure or recurrence of tuberculosis. The results of this study revealed significant improvements in clinical and imaging outcomes in both the NSBG and SBG groups, and the NSBG had less procedure time, bone fusion time, and intraoperative blood loss. Therefore, we believe that using NSBGs in selected cases is safe and effective, and spinal stability plays a key role in determining the style of the bone graft. In previous clinical practice, NSBG was selected under the following conditions: newly formed bone bridging the affected vertebra, at least one side of the lateral wall of the vertebral body was intact; at least one side of the pedicle was intact for placing a pedicle screw; the local Cobb angle was less than 20°; and the bone defect was less than half the height of the vertebral body. However, these criteria cannot be accurately quantified, and generally accepted standards for choosing NSBG or SBG should be developed. Moreover, developing a simple, easy-to-identify, reliable, and effective standard can help select appropriate treatment methods and facilitate peer communication and scientific research [22]. Spinal stability is a crucial factor when choosing the procedure for many spinal disorders, including spinal tuberculosis. In addition to debridement and anti-tuberculosis medication, restoring and maintaining spinal stability is important for controlling tuberculosis infection. Instability of the spine is defined as the loss of spinal integrity due to a pathological change with motion-associated pain and progressive deformity, with or without nerve defects [23]. So far, there are few reports on scenarios of judging the spinal stability of a tuberculosis-infected spine and directing the choice of bone graft material [24, 25]. Spinal tuberculosis has clinical manifestations that are similar to those of spinal metastasis, including back pain, weight loss, weakness, and even neurological dysfunction, as well as imaging features, such as vertebral destruction, pathological fracture, and kyphosis deformity [26]. In this study, we applied the SINS to evaluate the stability of the spine and expected to develop a quantitative standard for guiding the selection of bone grafts in patients with tuberculosis of the thoracolumbar spine.

First published in 2010, the SINS was originally developed as the standard for assessing the level of spinal stability in patients with spinal tumors. It including six items (posterolateral involvement of spinal elements, vertebral body collapse, spinal alignment, bone lesion quality, mechanical pain, and lesion location) and had a maximum score of 18 [9].

This research has some limitations. First, the number of patients included in this retrospective study was small. Prospective research at multiple centers with more cases should be performed in the future. Second, spinal tuberculosis is intrinsically different from spinal metastasis in the style of bone destruction, as hyperostosis and bone bridges frequently occur in spinal tuberculosis. To develop an accurate evaluation system for spinal tuberculosis, the bone destruction score should be modified according to studies with more spinal tuberculosis cases. However, the present study provides a reference standard for choosing bone graft materials for cases of spinal tuberculosis.

## Conclusion

NSBGs and SBGs can achieve comparable therapeutic effects in patients with spinal tuberculosis, and a suitable selection of bone grafts based on quantitative SINS will make full use of the advantages of different bone grafts.

## Data Availability

The datasets used and/or analysed during the current study available from the corresponding author on reasonable request.

## Abbreviations

ASIA: American Spinal Injury Association
CRP: C-reactive protein
ESR: Erythrocyte sedimentation rate
NSBG: Non-structural bone graft group
ODI: Oswestry disability index
SBG: Structural bone graft group
SINS: Spinal instability neoplastic score
VAS: Visual analog scale
